# Spectrum of Mutations in Pediatric Non-glomerular Chronic Kidney Disease Stages 2–5

**DOI:** 10.3389/fgene.2021.697085

**Published:** 2021-07-06

**Authors:** Xiaoyuan Wang, Huijie Xiao, Yong Yao, Ke Xu, Xiaoyu Liu, Baige Su, Hongwen Zhang, Na Guan, Xuhui Zhong, Yanqin Zhang, Jie Ding, Fang Wang

**Affiliations:** Department of Pediatrics, Peking University First Hospital, Beijing, China

**Keywords:** non-glomerular, chronic kidney disease, renal hypodysplasia, cystic kidney diseases, targeted exome sequencing, genetic diagnosis

## Abstract

Renal hypodysplasia and cystic kidney diseases, the common non-glomerular causes of pediatric chronic kidney disease (CKD), are usually diagnosed by their clinical and imaging characteristics. The high degree of phenotypic heterogeneity, in both conditions, makes the correct final diagnosis dependent on genetic testing. It is not clear, however, whether the frequencies of damaged alleles vary among different ethnicities in children with non-glomerular CKD, and this will influence the strategy used for genetic testing. In this study, 69 unrelated children (40 boys, 29 girls) of predominantly Han Chinese ethnicity with stage 2–5 non-glomerular CKD caused by suspected renal hypodysplasia or cystic kidney diseases were enrolled and assessed by molecular analysis using proband-only targeted exome sequencing and array-comparative genomic hybridization. Targeted exome sequencing discovered genetic etiologies in 33 patients (47.8%) covering 10 distinct genetic disorders. The clinical diagnoses in 13/48 patients (27.1%) with suspected renal hypodysplasia were confirmed, and two patients were reclassified carrying mutations in nephronophthisis (*NPHP*) genes. The clinical diagnoses in 16/20 patients (80%) with suspected cystic kidney diseases were confirmed, and one patient was reclassified as carrying a deletion in the hepatocyte nuclear factor-1-beta gene (*HNF1B*). The diagnosis of one patient with unknown non-glomerular disease was elucidated. No copy number variations were identified in the 20 patients with negative targeted exome sequencing results. *NPHP* genes were the most common disease-causing genes in the patients with disease onsets above 6 years of age (14/45, 31.1%). The children with stage 2 and 3 CKD at onset were found to carry causative mutations in paired box gene 2 (*PAX2*) and *HNF1B* gene (11/24, 45.8%), whereas those with stage 4 and 5 CKD mostly carried causative mutations in *NPHP* genes (19/45, 42.2%). The causative genes were not suspected by the kidney imaging patterns at disease onset. Thus, our data show that in Chinese children with non-glomerular renal dysfunction caused by renal hypodysplasia and cystic kidney diseases, the common causative genes vary with age and CKD stage at disease onset. These findings have the potential to improve management and genetic counseling of these diseases in clinical practice.

## Introduction

The presence of structural or functional abnormalities in the kidney over a 3-month period is defined as chronic kidney disease (CKD), and is classified into five stages based on the glomerular filtration rate (Andrassy, 2013). End-stage renal disease (ESRD), which is the most serious CKD stage, requires the use of renal replacement therapy. Pediatric CKDs are less common than in adults, but affected children are at increased risk of early mortality and disabling physical comorbidities, which highlights the need for appropriate management of the affected children.

Congenital anomalies of the kidney and urinary tract (CAKUT) are the most common non-glomerular presentations of pediatric ESRD followed by cystic kidney diseases (Smith et al., 2007; Wuhl et al., 2014). In fact, the most prevalent malformation is reported to be renal hypodysplasia, which includes renal aplasia, hypoplasia, and dysplasia (Smith et al., 2007). Renal ultrasound provides essential diagnostic information about renal hypodysplasia and cystic kidney diseases (Sanna-Cherchi et al., 2007; Vester et al., 2010; Gimpel et al., 2019). For example, hypodysplastic kidney is defined by renal ultrasonography findings as a reduced renal size of greater than two standard deviations from the mean size in terms of age and loss of corticomedullary differentiation, and the sonographic signs of parenchymal hyperechogenicity and renal enlargement in a child are highly suggestive of polycystic kidney disease. However, a clinically definitive diagnosis of hypodysplastic kidney disease or cystic kidney disease remains challenging to arrive at because the sonographic appearance of these two conditions is observed in a variety of renal diseases.

With advances in genomic DNA sequencing technologies, the genetic mechanisms leading to renal hypodysplasia and cystic kidney diseases have been more readily assessed (Vivante and Hildebrandt, 2016; Sanna-Cherchi et al., 2018; Armstrong and Thomas, 2019; Devlin and Sayer, 2019; Nigam et al., 2019), and this improves the diagnostic accuracy of genetic testing. Our previous study reported that the genetic test results for pediatric steroid-resistant nephrotic syndrome vary by ethnicity (Wang et al., 2017b). It is not clear, however, whether a similar phenomenon exists with the pediatric chronic renal dysfunction caused by renal hypodysplasia and cystic kidney diseases, which provides the impetus for the reasonable selection of genetic testing approaches. To address this question, index-only targeted exome sequencing and array-comparative genomic hybridization (CGH) were performed in a cohort of 69 unrelated children with non-glomerular stage 2–5 CKD who were clinically suspected of having renal hypodysplasia or cystic kidney diseases.

## Materials and Methods

### Patients

Patients were enrolled in the study between January 2011 and September 2018 by a group of trained pediatric nephrologists from the Department of Pediatrics, Peking University First Hospital based on fulfillment of the following criteria: (i) the presence of stage 2–5 CKD below the age of 18 years; (ii) clinical diagnosis or suspicion of renal hypoplasia/dysplasia, cystic kidney diseases, or unknown non-glomerular diseases. Patients with polycystic kidney disease, incomplete clinical data (especially the absence of kidney imaging results), and an unwillingness to participate in the study were excluded. The study was approved by the Ethics Committee of Peking University First Hospital and was performed in accordance with the Declaration of Helsinki.

Comprehensive clinical data [including age of onset, age of renal failure, urinalysis, examination of urinary protein, renal imaging, estimated glomerular filtration rate using 24-h endogenous creatinine clearance or the Schwartz formula (Schwartz et al., 1987), extrarenal manifestations, renal biopsy, information from the last follow-up, and family history] and demographics were extracted from the Chinese Registry Database of Hereditary Kidney Diseases and then analyzed. Sonographic measurements of the longitudinal sections of both kidneys in each patient were compared with those of age-matched controls (Loftus et al., 1998).

After receiving informed consent from the patients or their parents/legal guardians, blood samples and comprehensive clinical data were collected and analyzed.

### Genetic Examination

Genomic DNA was extracted from peripheral white blood cells using the QIAamp DNA Blood Mini Kit (A1120, Qiagen, Germany). DNA quantity and quality were determined by NanoDrop (Thermo Fisher Scientific, United States). When available, DNA samples from the participants’ relatives were obtained.

Because targeted exome sequencing is a cost-effective diagnostic strategy for identifying the genetic causes of kidney disorders (Vivante and Hildebrandt, 2016; Groopman et al., 2018), we used it to simultaneously examine 30 genes that are known to be associated with renal hypodysplasia and 118 genes associated with cystic kidney diseases (Supplementary Table 1). These genes were selected from the relevant literature (Devuyst et al., 2014; Mann et al., 2019; Nigam et al., 2019). DNA library preparation, capture, enrichment, next-generation sequencing, and data analysis were performed at BGI-Shenzhen, China, as described previously (Wang et al., 2017b). Variants with minor allele frequencies <0.01 were selected based on the control database such as NCBI dbSNP (snp137), 1000 Genomes Project (phase I), Exome sequencing project (ESP6500), Exome Aggregation Consortium (ExAC), Genome Aggregation Database (gnomAD), and the BGI in-house database. The Human Gene Mutation Database (HGMD) and ClinVar were used to detect previously reported pathogenic variants. The prioritized variants were classified according to the American College of Medical Genetics and Genomics (ACMG) guidelines (Richards et al., 2015).

To detect copy number variations (CNVs), array CGH was performed using the Agilent SurePrint G3 Human 8 × 60 K CGH Microarray (Agilent Technologies, Technologies, Santa Clara, CA, United States). DNA labeling, array hybridization, scanning, and data analysis were conducted at the Department of Central Laboratory, Peking University First Hospital, Beijing, China, as described previously (Yi et al., 2016). Public CNV databases including DGV, NCBI, DECIPHER, ClinGen, OMIM, and ISCA were used to detect known CNVs. The prioritized CNVs were classified according to the ACMG guidelines (Riggs et al., 2020).

Validation of all candidate pathogenic or likely pathogenic variants was performed using Sanger sequencing or quantitative PCR (qPCR) on the genomic DNAs of the probands. Hepatocyte nuclear factor-1-beta gene (*HNF1B*) and the nephronophthisis type 1 (*NPHP1*) gene were used to normalize the gene dosage in qPCR, and they were analyzed in triplicate. Segregation analyses were performed for all the available first-degree relatives.

## Results

### Clinical Features

**FIGURE 1:**
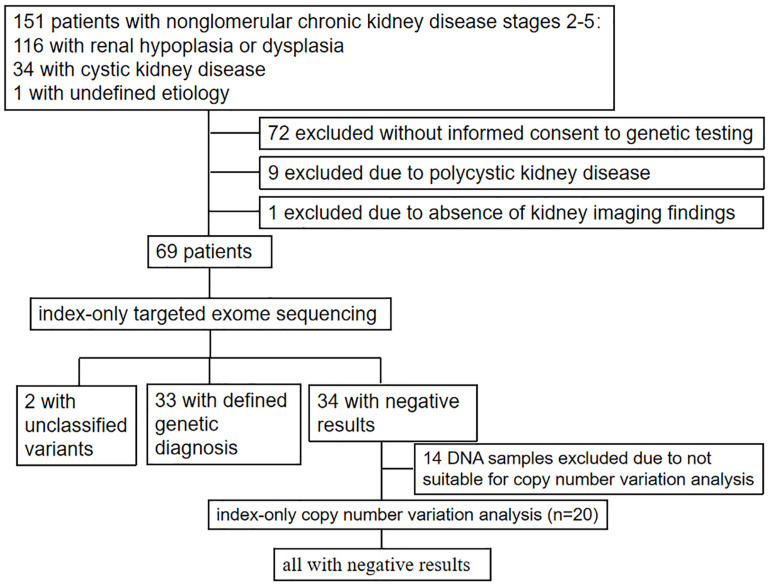
Flow diagram for patient selection and genetic testing.

As shown in Figure 1 and Table 1, 69 unrelated patients (40 boys, 29 girls) were enrolled in this study. They were from 18 provinces, municipalities, and autonomous Chinese regions and were predominantly the Han Chinese ethnicity (61/69). Renal dysfunction was detected in most of these patients (36/69), either accidentally or for other reasons at disease onset, whereas complaints of fatigue or a sallow complexion were observed in 19 patients, edema in eight patients, short stature in four patients, polydipsia and polyuria in one patient, and enuresis in another patient. Their median age at disease onset was 8.5 years (range, 0 day–16.7 years). Renal hypoplasia/dysplasia, cystic kidney disease, and unknown non-glomerular disease were diagnosed or suspected in 48, 20, and 1 patient, respectively. There were seven patients with stage 2 CKD, 17 patients with stage 3 CKD, 16 patients with stage 4 CKD, and 29 patients with stage 5 CKD. Of the 17 patients undergoing renal biopsy, chronic tubulointerstitial nephropathy was the most common histopathological diagnosis. The patients’ extrarenal manifestations included short stature, ocular abnormalities (including ametropia, strabismus, microphthalmia, retinopathy, vitreous opacity, and nystagmus), auricle malformation, preauricular fistula, spina bifida, cryptorchidism, skeletal deformities (including polydactylism, tetradactylism, straw sandal-like feet, strephenopodia, and fourth metatarsal microsomia), elevated liver enzymes, ovarian teratoma, microcephaly, ventricular septal defect, and patent arterial duct in 23 patients. Parental consanguinity was reported in only one patient, whereas eight patients had positive family histories of ESRD, one patient had a positive family history of proteinuria, and one patient had a positive family history of renal cystic disease. Eight patients had received a renal transplant (median age, 12.9 years; range, 6–18 years), and no disease recurrence in their allografts was documented. Six patients died at a median age of 5.9 (2–12) years.

**TABLE 1:** Clinical features of the 69 patients from the present study.

Parameter	Patients with molecular diagnosis* (*n* = 35)	Patients without molecular diagnosis (*n* = 34)
**Gender (M, F)**	15, 20	25, 9
**Age of onset, years**	9.4 (0–16.7)	8.1 (0–15.0)
**Follow-up time, months**	27 (7–120)	17 (3–120)
**Age of genetic test, years**	10.0 (1.0–16.7)	8.8 (0.2–16.0)
**Clinical diagnosis** Renal hypoplasia/dysplasia Cystic kidney disease Unknown non-glomerular disease	25 9 1	23 11 0
**CKD stage at disease onset** (CKD stage 2, 3, 4, 5, *n*) <1 year 1−3 years 3−6 years 6−12 years 12−18 years	1, 0, 1, 1 0, 2, 2, 1 1, 2, 0, 2 1, 2, 5, 7 1, 1, 3, 2	0, 1, 1, 2 0, 1, 2, 1 0, 1, 1, 2 2, 6, 1, 6 1, 1, 0, 5
**Extrarenal manifestations**	14	9
**Renal histopathologic diagnosis**		
Chronic tubulointerstitial nephropathy with or without glomerular lesions	10	2
Oligomeganephronia with atypical membranous nephropathy	1	0
Oligomeganephronia?	0	1
Focal proliferative sclerosing purpura nephritis with glomerular hypertrophy	1	0
Focal segmental glomerular sclerosis	1	1
**Family history**	5	5

*Including two cases carrying putatively pathogenic *NPHP3* variants that required further functional verification.

### Genetic Study

**FIGURE 2:**
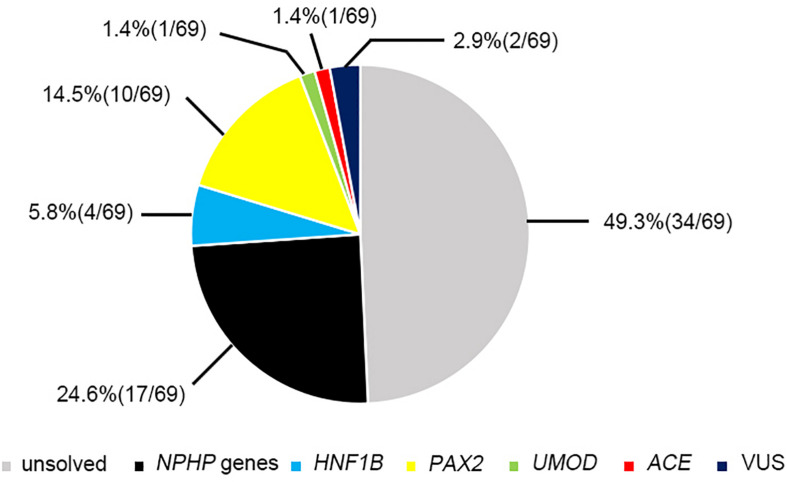
The results of genetic examination in 69 patients.

Twenty-two pathogenic variants and six likely pathogenic variants in 10/148 targeted genes, including nine non-sense, seven missense, six splice sites, three small deletions, two whole gene deletion, and one small insertion, were detected in 33/69 patients (47.8%), and these variants encompassed 10 distinct genetic disorders (Table 2 and Figure 2). Of these variants, the 14 (50.0%) novel ones included three variants that we reported on previously (Deng et al., 2019), whereas the remaining 14 variants were previously reported. Of the 18 patients harboring diagnostic variants in recessive genes, compound heterozygous variants were found in 10 patients and homozygous variants were found in eight patients. Of the 48 patients with suspected renal hypodysplasia, the targeted exome sequencing confirmed the clinical diagnoses of 13 patients (27.1%), and reclassified the clinical diagnoses of two patients carrying mutations in nephronophthisis (NPHP) genes (*INVS* and *WDR19*). Of the 20 patients with suspected cystic kidney diseases, the clinical diagnoses for 16 patients (80%) were confirmed, and that of one patient with a deletion in *HNF1B* was reclassified. The diagnosis of the remaining patient (patient 19) with renal dysfunction (serum creatinine, 122 μmol/L; evaluated glomerular filtration rate, 22.9 ml/min/1.73 m^2^), moderate anemia (70 g/L), short stature (height, 71 cm), and normal-sized, non-cystic kidneys combined with parenchymal hyperechogenicity and poor corticomedullary differentiation on renal ultrasonography (11 months of age) was classified by the compound heterozygous non-sense mutations present in the gene encoding the angiotensin-converting enzyme (*ACE*).

**TABLE 2:** Pathogenic or likely pathogenic variants detected by targeted exome sequencing*.

Patient ID	Gender	Age at onset	Clinical diagnosis	Renal ultrasound findings	Renal biopsy (at age)	Extrarenal manifestations	Follow-up (at age)	Gene	Nucleotide alteration	Genomic position and SNP	Amino acid changes	Location (zygosity, segregation)	ACMG classify sequence variants	ACMG interpretation	Ref.
19	Female	11M	Unknown non-glomerular diseases, CKD4	Normal size kidneys without cyst	ND	NO	Loss to follow-up	*ACE*	c.793C >T	g.61557835C >T (rs138873311)	p.Arg265*	EX5 (het, mother)	PVS1 PM2 PP3 PP4	Pathogenic	Gribouval et al., 2012
								*ACE*	c.1028G >A	g.61559009G >A (rs11466112)	p.Trp343*	EX7 (het, paternal)	PVS1 PM2 PP3 PP4	Pathogenic	Gribouval et al., 2012
36	Male	3Y	Renal hypoplasia/dysplasia, CKD3	Small size kidneys without cyst	ND	NO	Loss to follow-up	*HNF1B*	EX1-9del	–	–	The whole gene (het, *de novo*)	PVS1 PS2 PM2 PP4 PP3	Pathogenic	Weber et al., 2006
4	Female	11Y	Cystic kidney diseases, CKD4	Normal size kidneys with a cyst	ND	NO	Transplant (15Y)	*NPHP1*	EX1-20 del	–	–	The whole gene (hom, ?)	PVS1 PM2 PP3 PP4	Pathogenic	Kang et al., 2016
11	Female	2Y	Renal hypoplasia/dysplasia, CKD4	Small size kidneys without cyst	Chronic tubulointerstitial nephropathy	NO	PD (17Y)	*NPHP1*	EX1-20 del	–	–	The whole gene (hom, ?)	PVS1 PM2 PP3 PP4	Pathogenic	Kang et al., 2016
14	Female	6Y4M	Renal hypoplasia/dysplasia, CKD5	Small size kidneys without cyst	ND	NO	CKD5 (10Y)	*NPHP1*	EX1-20 del	–	–	The whole gene (hom, maternal)	PVS1 PM2 PP3 PP4	Pathogenic	Kang et al., 2016
20	Female	11Y6M	Renal hypoplasia/dysplasia, CKD5	Small size kidneys with a cyst	ND	NO	Transplant (12Y7M)	*NPHP1*	EX1-20 del	–	–	The whole gene (hom, paternal, maternal)	PVS1 PM2 PP3 PP4	Pathogenic	Kang et al., 2016
27	Male	13Y6M	Renal hypoplasia/dysplasia, CKD4	Small size kidneys without cyst	ND	Short stature	Loss to follow-up	*NPHP1*	EX1-20 del	–	–	The whole gene (hom, maternal)	PVS1 PM2 PP3 PP4	Pathogenic	Kang et al., 2016
33	Male	9Y	Cystic kidney diseases, CKD5	Normal size kidneys with cysts	ND	Astigmatism, strabismus	CKD5 (10Y)	*NPHP1*	EX1-20 del	–	–	The whole gene (hom, ?)	PVS1 PM2 PP3 PP4	Pathogenic	Kang et al., 2016
37	Male	13Y2M	Cystic kidney diseases, CKD5	Normal size kidneys with a cyst	Chronic tubulointerstitial nephropathy	NO	PD (13Y9M)	*NPHP1*	c.1122+4 delA	g.110919176delT	–	IVS10 (het, maternal)	PVS1 PM2 PP3 PP4	Pathogenic	This report
								*NPHP1*	EX1-20 del	–	–	The whole gene (het, paternal)	PVS1 PM2 PP3 PP4	Pathogenic	Kang et al., 2016
52	Female	16Y8M	Cystic kidney diseases, CKD4	Normal size kidneys without cyst	ND	NO	Loss to follow-up	*NPHP1*	EX1-20 del	–	–	The whole gene (hom, paternal, maternal)	PVS1 PM2 PP3 PP4	Pathogenic	Kang et al., 2016
9	Female	3Y9M	Cystic kidney diseases, CKD5	Normal size kidneys with cysts	ND	NO	Transplant (6Y)	*INVS (NPHP2)*	c.2782C >T	g.103055321C > T(rs376879175)	p.Arg928*	EX14 (het, paternal)	PVS1 PM2 PP3 PP4	Pathogenic	Halbritter et al., 2013
								*INVS (NPHP2)*	c.2666_2667 delTG	g.103055205_10 3055206delTG	p.Val889Glufs*3	EX14 (het, maternal)	PVS1 PM2 PP3 PP4	Pathogenic	This report
26	Male	11M	Renal hypoplasia/dysplasia, CKD5	Small size kidneys without cyst	Chronic tubulointerstitial nephropathy with glomerular lesions	NO	Died (2Y)	*INVS (NPHP2)*	c.2701C >T (het)	g.1030552 40C >T	p.Gln901*	EX14 (het, maternal)	PVS1 PM2 PP3 PP4	Pathogenic	This report
								*INVS (NPHP2)*	c.2786+2T >C	g.103055327T > C(rs1322951938)	-	IVS14 (het, paternal)	PVS1 PM2 PP3 PP4	Pathogenic	Otto et al., 2003
60	Male	6Y	Renal hypoplasia/dysplasia, CKD5	Small size kidneys without cyst	ND	NO	CKD5 (7Y)	*NPHP3*	c.909C >A	g.132433977G >T	p.Tyr303*	EX5 (het, paternal)	PVS1 PM2 PP3 PP4	Pathogenic	This report
								*NPHP3*	c.3202-2A >G	g.1324052 33T >C		IVS22 (het, maternal)	PVS1 PM2 PP3 PP4	Pathogenic	This report
2	Male	9Y9M	Renal hypoplasia/dysplasia, CKD5	Small size kidneys with cysts	ND	Cryptorchidism	Died (12Y)	*NPHP3*	c.1082C > G (het)	g.132432006G >C (rs146250226)	p.Ser361Cys	EX6 (het, maternal)	PM1 PM2 PP3 PP4	Likely pathogenic	ClinVar
								*NPHP3*	c.1986-2A >G	g.132416208T >C	–	IVS13 (het, paternal)	PVS1 PM2 PP3 PP4	Pathogenic	This report
61	Female 11Y cystic kidney diseases, CKD4 normal size kidneys with cysts MPGN, tubulointerstitial histopathology growth retardation CKD5(13y)	11Y	Renal hypoplasia/dysplasia, CKD	Normal size kidneys with cysts	MPGN, tubulointerstitial histopathology	Growth retardation	CKD5 (13Y)	*NPHP4*	c.992+1G >A	g.6008129C >T	–	IVS8 (het, paternal)	PVS1 PM2 PP3 PP4	Pathogenic	This report
								*NPHP4*	c.2260G >A	g.5950972C >T (rs373962831)	p.Gly754Arg	EX17 (het, maternal)	PM1 PM2 PP3 PP4	Likely pathogenic	Otto et al., 2002
1	Female	14Y	Renal hypoplasia/dysplasia, CKD5	Small size kidneys with a cyst	Chronic tubulointerstitial nephropathy	Nystagmus, hypermetropia, astigmatism	Loss to follow-up	*IQCB1 (NPHP5)*	c.1090C >T	g.121508959G >A (rs727503968)	p.Arg364*	EX11 (het, paternal)	PVS1 PM2 PP3 PP4	Pathogenic	Khanna et al., 2009
								*IQCB1 (NPHP5)*	c.1333C >T	g.121500667G >A	p.Arg445*	EX13 (het, maternal)	PVS1 PM2 PP3 PP4	Pathogenic	Halbritter et al., 2012
63	Female	8Y6M	Renal hypoplasia/dysplasia, CKD5	Small size kidneys without cyst		NO	Loss to follow-up	*WDR19*	c.641T >A	g.39206811T >A (rs751290509)	p.Leu214*	EX8 (het, maternal)	PVS1 PM2 PP3 PP4	Pathogenic	VKGL-NL_AMC^$^
								*WDR19*	c.904G >T	g.39216234G >T	p.Asp302Tyr	EX10 (het, *de novo*)	PM1 PM2 PM3 PP3 PP4	Pathogenic	This report
16	Female	6Y9M	Cystic kidney diseases, CKD4	Normal size kidneys with cysts	ND	NO	CKD5 (10Y9M)	*UMOD*	c.178G >T	g.20360445C >A	p.Gly60Cys	EX3 (het, *de novo*)	PS2 PM1 PM2 PP3	Likely pathogenic	This report

*We assessed the Human Gene Mutation Database and the Leiden Open Variation Database (LOVD) to check novel variants in April 2021. Nucleotide positions are numbered in accordance with the reference sequences (NM_000789.3 for *ACE*, NM_000458.4 for *HNF1B*, NM_003990.3 for *PAX2*, NM_000272.3 for *NPHP1*, NM_014425.3 for *INVS*, NM_153240.4 for *NPHP3*, NM_015102.3 for *NPHP4*, NM_001023570.2 for *IQCB1*, NM_025132.3 for *WDR19*, and NM_003361.2 for *UMOD*) using the first coding ATG of exon 1 as the initiation codon. Patients 8, 10, 13, 18, 21, 24, 30, 32, 34, and 38 have already been reported (Deng et al., 2019). Patients 6, 28, and 29 have also been reported (Wang et al., 2017a). Patients 23 and 31 have also been reported (Deng et al., 2020). ^$^LOVD. ACMG, the American College of Medical Genetics and Genomics; CKD, chronic kidney disease; ND, not done; PD, peritoneal dialysis; IVS, intron; EX, exon; Hom, homozygous; Het, heterozygous; M, months; Y, years; PVS, pathogenic very strong; PS, pathogenic strong; PM, pathogenic moderate; PP, pathogenic supporting.

Patients 22 and 69 were strongly suspected of having NPHP based on the combination of ESRD before the age of 7 years without proteinuria or hematuria, normal-sized kidneys with hyperechogenicity and the absence of corticomedullary differentiation, and chronic tubulointerstitial nephropathy (in patient 22) or elevated liver enzymes of unknown cause (in patient 69), whereas targeted exome sequencing revealed three rare and predicted deleterious variants in *NPHP3* that were classified as having unknown significance using ACMG criteria (Table 3). We assumed that these variants are pathogenic, although functional analyses on them are required.

**TABLE 3:** Variants of unknown significance that were detected by targeted exome sequencing.

Patient ID	Gender	Age at onset	Clinical diagnosis	Renal ultrasound findings	Renal biopsy (at age)	Extrarenal manifestations	Follow-up (at age)	Gene	Nucleotide alteration	Genomic position and SNP	Deduced amino acid changes	Location (zygosity, segregation)	ACMG classify sequence variants	ACMG interpretation	Ref.
22	Female	2Y2M	Cystic kidney diseases, CKD5	Normal size kidneys without cysts	Chronic tubulointerstitial nephropathy	Short stature	CKD5 (6Y6M)	*NPHP3*	c.3813-3A >G	g.132400937T >C	–	IVS26 (hom, mother)	PM2 PP3 PP4	Unknown significance	This report
69	Male	3Y	Cystic kidney diseases, CKD5	Normal size kidneys with cysts	FSGS	Abnormal liver function	PD (3Y8M)	*NPHP3*	c.3813-3A >G	g.132400937T >C	–	IVS26 (het, father)	PM2 PP3 PP4	Unknown significance	This report
								*NPHP3*	c.1135T >C	g.132427085A >G	p.Cys379Arg	EX7 (het, mother)	PM2 PP3 PP4	Unknown significance	This report

*Accession no.: *NPHP3*, NM_153240.4. ACMG, the American College of Medical Genetics and Genomics; CKD, chronic kidney disease; FSGS, focal segmental glomerulosclerosis; PD, peritoneal dialysis; IVS, intron; EX, exon; Hom, homozygous; Het, heterozygous; PM, pathogenic moderate; PP, pathogenic supporting.*

No CNVs were identified in the 20 patients with negative targeted exome sequencing results.

**FIGURE 3:**
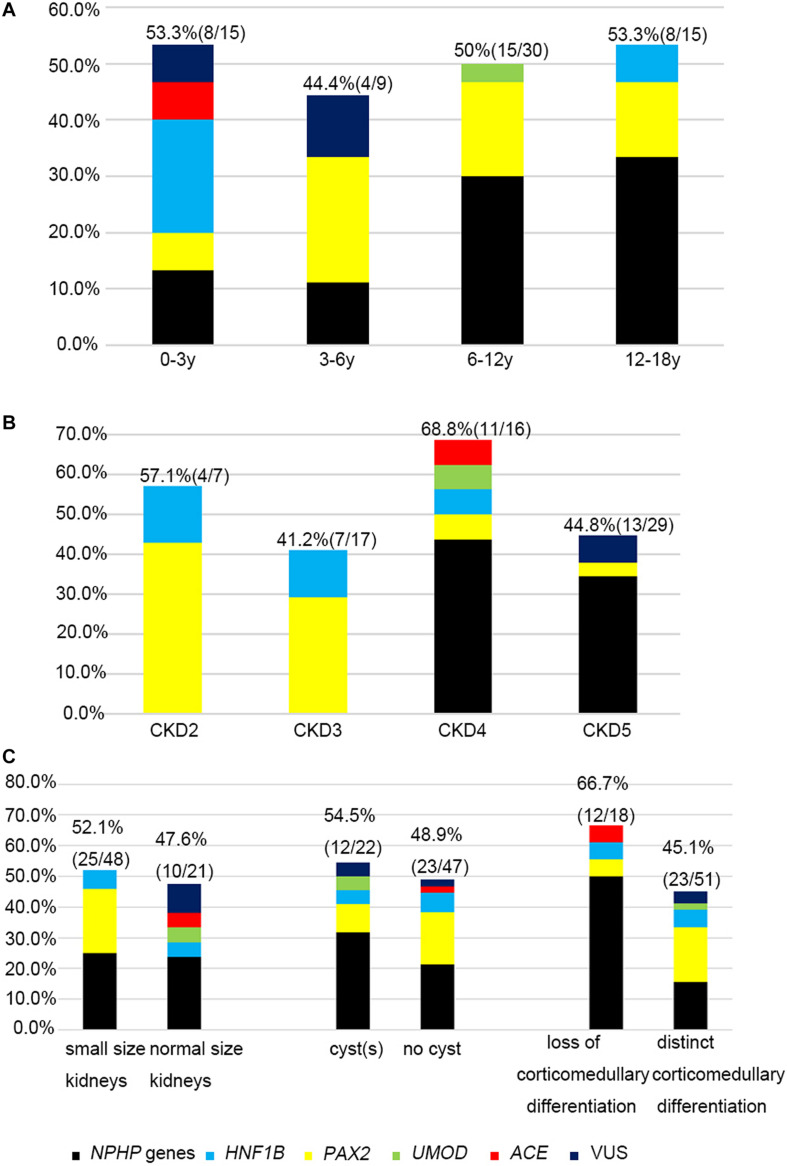
The relationship between causative genes and clinical features. Histograms indicate fractions (in percentage) of patients with disease gene detected per group. **(A)** Genetic diagnosis and the age at onset for non-glomerular CKD. **(B)** Genetic diagnosis and CKD stages. **(C)** Genetic diagnosis and renal ultrasound findings.

The likelihood of establishing an accurate molecular diagnosis of non-glomerular CKD did not improve with increasing age and remained roughly the same (at about 50%) (Figure 3A). We detected diagnostic *PAX2* and *NPHP* gene variants in all four age groups, and the *NPHP* genes were the most common disease-causing ones in the patients whose disease onset was above 6 years of age (14/45, 31.1%).

The molecular diagnostic performance and common mutated genes differed in line with the increased CKD stage at disease onset (Figure 3B). The genetic diagnostic yield was highest in the patients with stage 4 CKD at onset (11/16, 68.7%). The children whose CKD onset was stage 2 and 3 carried mutations in *PAX2* and *HNF1B* genes (11/24, 45.8%), whereas those whose CKD onset was stage 4 and 5 mostly carried mutations in *NPHP* genes (19/45, 42.2%).

Because renal ultrasonography is used in the first instance to diagnose renal hypodysplasia and cystic nephropathies, we analyzed the renal imaging patterns at disease onset and the mutation detection rates in the patients (Figure 3C). An etiological diagnosis was found in 52.1% of the children with small kidneys (25/48), 47.6% with normal-sized kidneys (10/21), 48.9% without cysts (23/47), 54.5% with a single cyst or multiple cysts (12/22), 66.7% without corticomedullary differentiation (12/18), and 45.1% with distinct corticomedullary differentiation (23/51). Renal parenchymal hyperechogenicity was observed in all 69 patients.

**FIGURE 4:**
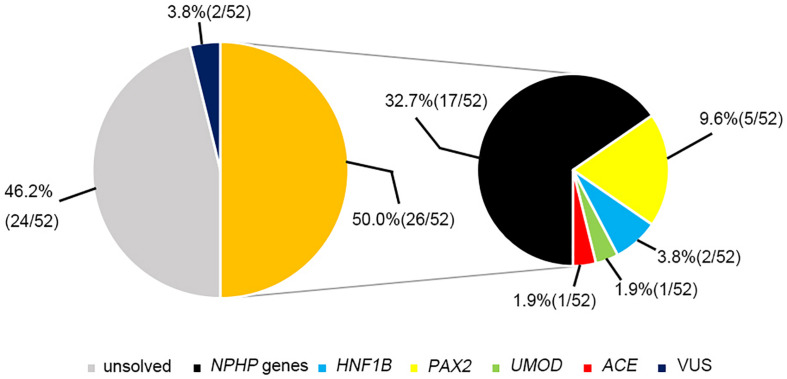
The genetic examination results for 52 patients who were highly suspected of having nephronophthisis.

The most prevalent genetic diagnosis in our study was NPHP. Chaki et al. (2011) reported that 100% of 440 patients with NPHP-related ciliopathies carried biallelic pathogenic variants in *NPHP* genes. We therefore analyzed the genetic test results from the patients who met at least one of the four criteria for NPHP used by Chaki et al. (2011). Hence, we were able to clinically diagnose 52 patients as having NPHP and found that 26 patients (50%) had pathogenic or likely pathogenic variants in the causative genes. Of these patients, 17 had *NPHP*, whereas we identified mutations in *PAX2*, *HNF1B*, *ACE*, and *UMOD* genes in the remaining patients (Figure 4).

## Discussion

In the present study, we used targeted exome sequencing and array CGH to depict the genetic features of 69 unrelated children with non-glomerular stage 2–5 CKD caused by suspected renal hypodysplasia or cystic kidney diseases. First, 27.1% of our patients with suspected renal hypodysplasia obtained a molecular diagnosis, and *PAX2* was the most common mutated gene (found in ten patients). In contrast, in one cohort of 159 Chinese CAKUT children (Rao et al., 2019), only four carried *PAX2* mutations. Ishiwa et al. (2019) performed genetic analysis on 66 Japanese patients with CAKUT (the number of patients with renal hypodysplasia is not available) and identified the etiologies in 14 individuals with renal hypodysplasia (21.2%). Ahn et al. (2020) identified the causative genes responsible for renal hypodysplasia in 12/76 Korean children (15.8%), and predominance of *HNF1B* mutations was seen in these patients. The mutation detection rates range from 7 to 17% in patients from Europe and the United States with renal hypodysplasia combined with or without renal failure (Weber et al., 2006; Thomas et al., 2011; Hwang et al., 2014; Nicolaou et al., 2016). The *SALL1* (spalt-like transcription factor 1) gene was detected more frequently in one cohort of patients (Hwang et al., 2014), whereas *PAX2* and *HNF1B* were detected in another cohort (Weber et al., 2006; Thomas et al., 2011; Nicolaou et al., 2016). One possible explanation for this discrepancy relates to the criteria used for selecting patients: our cohort contained patients with bilateral renal lesions, whereas other studies have contained patients with bilateral and unilateral renal hypodysplasia. Another possible explanation is the high genetic heterogeneity in this condition. Second, causative genes were identified in 85% of our patients with suspected cystic kidney diseases, and 10 patients carried *NPHP1* mutations, making it the most prevalent mutated gene. However, in other patient cohorts, about 70% of the children with cystic kidney diseases had monogenic disease, and the most frequent molecular diagnosis was autosomal recessive polycystic kidney disease or polycystic kidney disease (Bullich et al., 2018; Rao et al., 2019; Obeidova et al., 2020). Excluding polycystic kidney disease from our study may in part explain this discrepancy. Finally, a genetic diagnosis was obtained in three children where phenotypic overlapping caused the initial disease to be clinically misdiagnosed, one case of which had undiagnosed stage 4 CKD, which stresses the importance of genetic testing as one of the diagnostic workups in the pediatric CKD population.

It is worth noting the difference we observed for the common causative genes in relation to the age and CKD stage at disease onset. *NPHP* genes were the most frequently mutated genes in the patients whose onset exceeded 6 years of age with stage 4–5 CKD, whereas mutations in *HNF1B* and *PAX2* together were more prevalent in patients whose onset was less than 6 years of age and had become stage 2–3 CKD. These findings suggest that performing genetic testing in accordance with the age and CKD stage at disease onset may be an efficient strategy for the molecular diagnosis of children with non-glomerular CKD. In contrast, Weber’s study showed that *HNF1B* and *PAX2* mutations caused CKDs with an age of onset between 10 and 23 years (Weber et al., 2006), and autosomal recessive polycystic kidney disease was reported to be the most prevalent etiology in neonatal-onset cystic kidney diseases (Obeidova et al., 2020). The difference is likely impacted by the use of small populations of patients and the patients’ ethnic origins. Because early stage renal hypodysplasia and cystic kidney diseases are often clinically silent, patients with renal insufficiency who are usually detected accidentally may be referred for clinical diagnosis. Renal ultrasonography is currently the diagnostic mainstay. The presence of small-sized kidneys in a child always leads clinicians to make a diagnosis of renal hypodysplasia, and the presence of renal cysts support the diagnosis of cystic kidney disease. However, the phenotypic and genetic variability of these two conditions makes establishing the final clinical diagnosis challenging. As our study has shown, small- to normal-sized kidneys with or without cyst formation or changes in corticomedullary differentiation can be caused by mutant *NPHP* and *HNF1B* genes (Chaki et al., 2011; Avni et al., 2015), whereas mutations in *PAX2* lead to small-sized kidneys that often show distinct corticomedullary differentiation and no cysts (Bower et al., 2012).

*NPHP* is one of the most common inherited diseases leading to pediatric ESRD, and the phenotypes and genotypes in Chinese children with *NPHP* have been described (Tang et al., 2020; Yue et al., 2020). However, our finding shows that non-*NPHP* genes can also cause NPHP-like phenotypes, which emphasizes the difficulty in diagnosing NPHP in clinical settings. Similar phenomena have been reported elsewhere (Bullich et al., 2018; Mann et al., 2019).

Pathogenic CNVs, recognizably important etiological factors underlying renal hypodysplasia (Sanna-Cherchi et al., 2012; Verbitsky et al., 2019), are recommended to be detected by array-based technologies. However, consistent with prior reports on the use of targeted exome sequencing as a tool for identifying CNVs (Roberts et al., 2017; Ahn et al., 2020), our targeted exome sequencing and qPCR, we detected the deletion of the whole *HNF1B* and *NPHP1* genes in 4 and 10 patients, respectively, but no additional CNVs were identified using array CGH.

Obtaining a definite molecular diagnosis is very important for patients and their families and for facilitating genetic counseling. For example, *HNF1B* mutations are associated with diabetes mellitus (Clissold et al., 2015), and *NPHP1* genetic variants may cause multisystemic diseases and Joubert syndrome, among others (Soliman et al., 2012). Early discovery of related hidden symptoms and timely treatments are very important for patients.

We are conscious of some limitations in our study, which include the comparatively small patient cohort, the stringent clinical criteria for selecting patients, the absence of whole-exome sequencing in patients lacking a genetic diagnosis, and the lack of functional verification of novel unknown significance variants. False-negative results from targeted exome sequencing were possible in the patients with no detectable variants. Nonetheless, our study was performed in one of the largest referral centers on mainland China, and the patients were from 18 out of 34 provincial administrative Chinese regions, indicating that our study is somewhat representative of Chinese children with renal dysfunction caused by renal hypodysplasia and cystic kidney diseases, and our findings provide the genotypic features seen in them. To the best of our knowledge, this is the first cohort study to provide evidence about the association between causative mutations and the stage of CKD onset.

In summary, the Chinese children with non-glomerular renal dysfunction caused by renal hypodysplasia and cystic kidney diseases in this study that carried the common causative genes varied in the age and CKD stage at disease onset. This new knowledge should help with improving the management and genetic counseling of the abovementioned diseases in clinical practice.

## Data Availability Statement

The datasets for this article are not publicly available due to concerns regarding participant/patient anonymity. Requests to access the datasets should be directed to the corresponding authors.

## Ethics Statement

The studies involving human participants were reviewed and approved by the Ethical Committee of Peking University First Hospital approved the procedures in this study. Written informed consent to participate in this study was provided by the participants’ legal guardian/next of kin. Written informed consent was obtained from the individual(s), and minor(s)’ legal guardian/next of kin, for the publication of any potentially identifiable images or data included in this article.

## Author Contributions

FW and JD: conceptualization, formal analysis, writing – review and editing, visualization, supervision, project Administration, and funding acquisition. XW: methodology, software, investigation, and resources. HX, YY, KX, HZ, XL, BS, NG, XZ, and YZ: data curation. XW and FW: writing – original draft preparation. All authors contributed to the article and approved the submitted version.

## Conflict of Interest

The authors declare that the research was conducted in the absence of any commercial or financial relationships that could be construed as a potential conflict of interest.

## Supplementary Material

Supplementary Table 1 List of genes list associated with renal hypodysplasia and cystic kidney diseases.
